# The Integration Hypothesis: An Evolutionary Pathway to Benign SIV Infection

**DOI:** 10.1371/journal.ppat.0020015

**Published:** 2006-03-31

**Authors:** Viktor Müller, Rob J De Boer

Untreated human immunodeficiency virus (HIV) infection in humans is typically characterised by persistent high virus load, failure of the immune response to clear the virus, and fatal disease outcome. Natural hosts of closely related simian immunodeficiency viruses (SIVs)—e.g., sooty mangabeys [1,2]—maintain comparably high persistent virus levels and yet remain healthy. These contrasting observations have two important implications. First, the virulence (disease-causing ability) of HIV is not an inevitable consequence of its massive replication in the infected hosts. SIVsm, the virus of sooty mangabeys, can replicate to high levels without any discernible effect on the host. The prevalence of SIV infection among wild-living sooty mangabeys can exceed 50% [3], which is also consistent with nonpathogenic infection. The direct metabolic cost of virus production must therefore be small, and disease in HIV infection cannot be explained by a simple diversion of host resources to virus production. Second, sooty mangabeys do not avoid disease by controlling the virus to low levels. While humans with HIV and macaques experimentally infected with SIVsm mount a vigorous T cell response that can temporarily suppress virus replication, sooty mangabeys display hardly any T cell proliferation upon infection [4], and allow high levels of virus replication from the beginning of the infection. Instead of eliminating the direct effects of infection by suppressing the virus, they can apparently avoid the indirect effects that are responsible for disease in HIV infection.

The clinical progression of HIV infection is characterised by a gradual loss of CD4^+^ T cells, which constitute the primary target cell type of the virus and which are key players in adaptive immunity. Immune functions slowly decline, eventually giving rise to opportunistic infections that ultimately result in death in nearly all untreated cases. These processes were first thought to be driven by the loss of infected CD4^+^ T cells, killed directly by the cytopathic effect of the virus. However, accumulating evidence (reviewed in [5]) indicates that the aetiology of HIV infection involves a generalised chronic hyperactivation of the immune system, rather than direct cytopathic effects. In addition to infected CD4^+^ T cells, the turnover of uninfected CD4^+^ T cells, CD8^+^ T cells, and B cells is also elevated, and persistent activation is probably responsible for the dysfunction and apoptotic death of immune cells. The immediate decline of activation markers (e.g., the percentage of dividing CD4^+^ and CD8^+^ T cells [6], and the frequency of antibody-secreting cells [7]) after the initiation of effective antiretroviral therapy indicates that hyperactivation is induced by the virus itself, rather than by a homeostatic mechanism. Natural hosts of SIV, however, show hardly any sign of immune hyperactivation despite high virus levels, which may be their key to benign infection [2]. We here propose the “integration hypothesis”, claiming that the evolutionary pathway to peaceful coexistence may have involved the integration of retroviral elements into the germline of the hosts, and the subsequent expression of retroviral antigens during the maturation of host lymphocytes to induce partial tolerance to the host-specific viruses. Mammalian genomes have long been known to contain a large number of retrovirus-derived sequences [8], and HIV-1 proviral DNA has been found in the sperm cells of individuals who are infected [9], which demonstrates the possibility of germline integration. Natural hosts of SIV infections may thus have acquired germline-integrated SIV elements that could be expressed during the negative selection of T cells and/or B cells in the thymus [10] and the bone marrow [11], respectively. Once their antigen specificity has been determined by the rearrangement of their receptor genes, immature T cells spend about one to two weeks in the thymus [12], while immature B cells spend one to three days in the bone marrow [13], where they are exposed to contact with “self” antigens. Those cells that react to the presented antigens are deleted or anergised (or B cells can be rescued by changing their specificity by further receptor editing). The production of naïve lymphocytes continues throughout the lifetime of the individual (although thymic output decreases with age); therefore, negative selection also operates lifelong. This mechanism serves normally to remove lymphocytes that would react to self antigens, but would also operate on retrovirus-derived antigens expressed at the sites of negative selection. The thymic expression of the endogenous mouse mammary tumour virus has indeed been shown to induce tolerance [14], and retrovirus-transduced genes expressed by bone marrow–derived cells were able to inhibit B cell responses to the transduced genes in mice [15]. Alternatively, the thymic expression of SIV-derived antigens could generate regulatory T cells that could downregulate peripheral responses to these elements [16]. Both mechanisms would inhibit antigen-specific SIV-induced processes that contribute to chronic immune hyperactivation in nonnatural SIV and HIV infections.

Most germline retroviral integrations are probably purged from the genome during evolution. However, the thymus- and/or bone marrow–specific expression of those SIV antigens that are responsible for chronic hyperactivation would be beneficial for the host, and would therefore be conserved by natural selection. Considering that neonatal HIV infection by vertical transmission does not result in immune tolerance and inhibition of immune hyperactivation in humans, efficient tolerance induction probably requires the expression of a specific subset of viral antigens under appropriate conditions. Finally, efficient tolerance induction by host genomic elements depends on low variability of the targeted viral antigens. In the SIV hosts with long coevolutionary history, we therefore predict the thymic- and/or bone marrow–specific expression of conserved SIV-derived gene fragments. Importantly, immune responses against viral epitopes not involved in the induction of chronic immune hyperactivation are likely to be beneficial for the hosts and are therefore expected to be retained. Indeed, naturally infected sooty mangabeys do display humoral and cellular responses against SIVsm [17,18], although both responses appear to be weaker than the responses of SIVsm-infected macaques.

The integration hypothesis emphasises the role of host adaptation in host–SIV coevolution. Reducing indirect harm to the host is the mutual interest of the host and the virus, because it extends the lifespan of the host without limiting the replication of the virus. However, viral evolution towards decreasing virulence is probably limited by within-host competition: the lifespan of the host is expected to be determined by the most aggressive variant in the host [19]. Furthermore, the infection of macaques with SIVsm results in AIDS-like disease, accompanied by chronic immune hyperactivation, which suggests that the “benign” virus of sooty mangabeys has not evolved to avoid hyperactivation. In fact, several recent cross-species transmissions of SIVs to nonnatural hosts have resulted in pathogenic infections, including multiple transmissions of SIVsm to humans (giving rise to HIV-2 [20]) and Asian macaques [21], and multiple transmissions of SIV from chimpanzees to humans (giving rise to HIV-1 [22]) (Figure 1).

**Figure 1:**
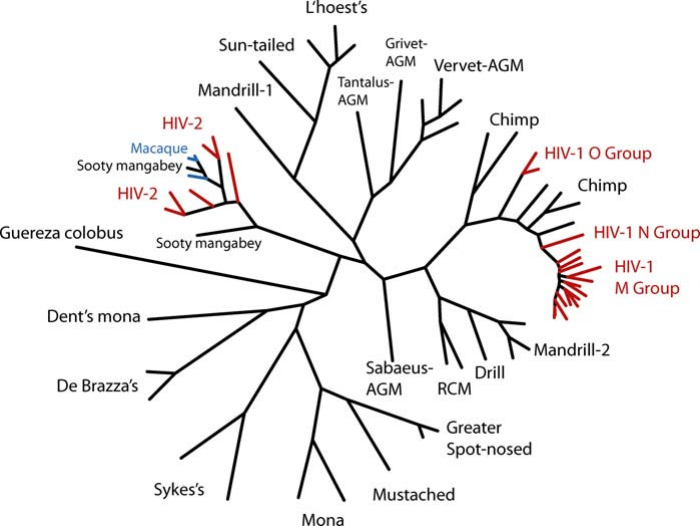
Evolutionary Relationships of Primate Lentiviruses Based on Maximum-Likelihood Phylogenetic Analysis of Full-Length Pol Protein Sequences Viruses responsible for natural infections of African primates are denoted by the name of the host species in black. Recent cross-species transmissions are shown in color: multiple transmissions from chimpanzees and sooty mangabeys have given rise to HIV infections in humans (red), and multiple transmissions from sooty mangabeys have given rise to nonnatural pathogenic SIV infections in rhesus, pig-tailed, and stumped-tailed macaques of Asian origin (blue). Aligned complete Pol sequences were downloaded from the Los Alamos database, and the phylogeny was generated with the PHYLIP package [23]. AGM, African green monkey; RCM, red-capped mangabey

At present, our hypothesis remains an intriguing possibility that requires experimental testing. It is not known if nonspecific mechanisms contribute to HIV-induced hyperactivation, which would limit the efficiency of antigen-specific tolerance induction. Whole-genome sequencing of the natural hosts of SIVs should demonstrate the presence of SIV-related sequences. In addition to actively expressed sequences involved in tolerance induction, silenced, nonexpressed gene fragments may also persist in the genome. Using predicted ancestral HIV-1 subtype B sequences (obtained from the Los Alamos database [http://hiv-web.lanl.gov/content/hiv-db/mainpage.html]) and SIVsm sequences (accession number M31325) of all viral proteins as queries, we performed translated similarity searches with tBLASTn against all sequences of primate origin in the complete “nonredundant” and expressed sequence tags databases of the National Center for Biotechnology Information Entrez Nucleotide database [24]. We found no genomic signatures of clearly lentiviral origin in any sequence derived from natural hosts of SIV infections. This negative result was to be expected because few sequences are currently available from the natural hosts of SIVs (chimpanzees are atypical SIV hosts with low virus loads and infection prevalence, probably more analogous to human long-term non-progressors). A more targeted approach would test a cDNA library of the cells involved in lymphocyte selection in natural hosts of SIVs. Finally, immune responses against the SIV-related antigenic patterns that are expressed in the host species should be difficult to elicit in the natural hosts due to immune tolerance. Successful breaking of this tolerance by boosted vaccines should in turn induce AIDS-like disease in infected animals.

Several years ago, Norley et al. [25] proposed that immunological tolerance to Gag could be responsible for avirulent SIV infection in natural hosts, and hypothesized that such tolerance could be achieved by the expression of endogenous retroviral elements. Our hypothesis has a different emphasis: we do not discuss specific mechanisms associated with particular viral genes, but focus on the possible evolutionary scenario that the origin of the endogenous elements may have been the very viruses that cause disease in the absence of tolerance.

The integration hypothesis proposes an evolutionary pathway to benign SIV infections that is technically plausible given the ability of retroviruses to insert their genetic material into the germline of the host, and that would not have been opposed by evolutionary barriers since it serves the mutual benefit of viruses and hosts. Whether this path was indeed taken by evolution must still be verified by experimental tests that can be performed by current technology. Whereas we have plenty of examples of viruses stealing host genes for their benefit, this would be a rare example of hosts utilising viral genes in the course of coevolution.

## Abbreviation

SIV: simian immunodeficiency virus
